# A large dedifferentiated retroperitoneal liposarcoma extended to the testis: a rare case report

**DOI:** 10.1093/jscr/rjad162

**Published:** 2023-05-29

**Authors:** Patrice Habonimana, Evrard Niyonkuru, Simon Nisabwe, Asmae Mazti, Amine Moataz, Mohamed Dakir, Adil Debbagh, Rachid Aboutaieb

**Affiliations:** Urology Department, Ibn Rochd University Hospital Center, Casablanca, Morocco; Pathology Department, Ibn Rochd University Hospital Center, Casablanca, Morocco; Pathology Department, Ibn Rochd University Hospital Center, Casablanca, Morocco; Pathology Department, Ibn Rochd University Hospital Center, Casablanca, Morocco; Urology Department, Ibn Rochd University Hospital Center, Casablanca, Morocco; Urology Department, Ibn Rochd University Hospital Center, Casablanca, Morocco; Urology Department, Ibn Rochd University Hospital Center, Casablanca, Morocco; Urology Department, Ibn Rochd University Hospital Center, Casablanca, Morocco

**Keywords:** Retroperitoneal liposarcoma, inguinoscrotal tumor, dedifferentiated liposarcoma

## Abstract

Liposarcomas are neoplasms of mesodermal origin representing less than 1% of all malignant tumors and 1 to 2% of urogenital lesions. Primary retroperitoneal liposarcomas extending into the inguinal canal are rare. We present the case of a large retroperitoneal liposarcoma invading the left testicle and its spermatic cord. It was diagnosed by imaging as a large mass that compresses surrounding abdominal structures and communicating with the inguinal canal. A surgical intervention consisting of en bloc resection of the tumor and the left testicle with its cord was performed by 2 routes, intercostal and inguinal. Histology showed a dedifferentiated liposarcoma, which is a rare entity with a high rate of malignancy and a poor prognosis. The treatment of choice is wide surgical resection with clear margins; chemotherapy and radiotherapy are less sensitive. The patient did not manifest any particular complaints during the first six months after surgery.

## INTRODUCTION

Retroperitoneal liposarcomas (RPLPS) are rare mesenchymal tumors. They account for 7.5–25% of all soft tissue sarcomas and 1–2% of urogenital lesions [1]. The retroperitoneal location of this tumor is rare, and its extension in the inguinal region with scrotal involvement is not usual [2]. Given the large size of the tumor and its invasion into adjacent organs, complete resection with negative margins constitutes a surgical challenge [3]. We report a case of large dedifferentiated retroperitoneal liposarcoma involving the testis and its spermatic cord.

## CASE REPORT

**Figure 1:**
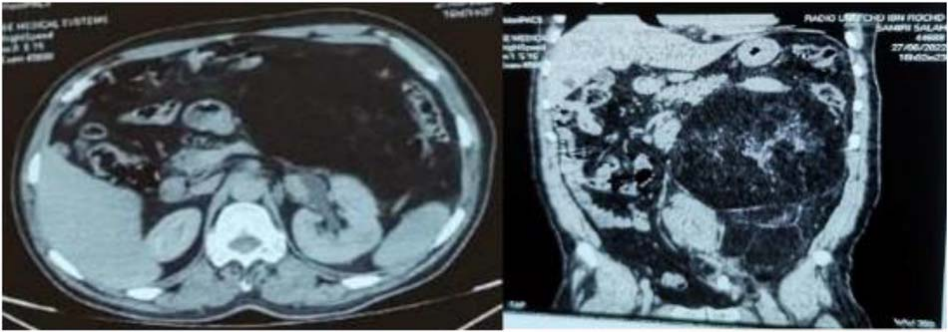
Axial and sagittal sections of an abdominal-pelvic CT scan showing a retroperitoneal liposarcoma compressing the surrounding structures and responsible for left pyelocaliceal dilation.

**Figure 2:**
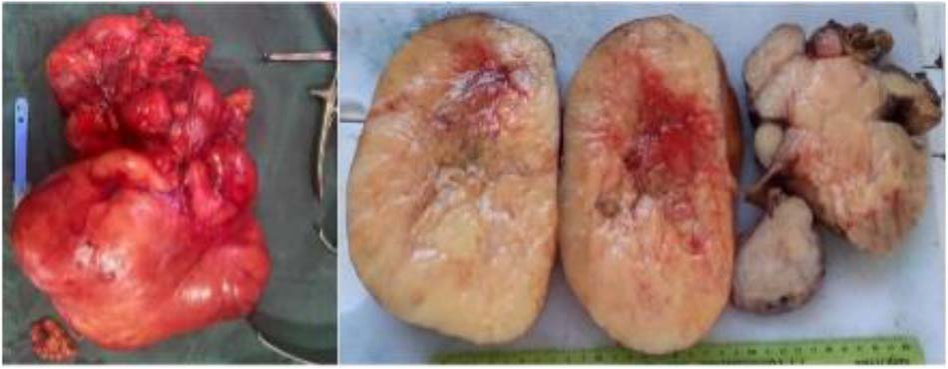
Macroscopic appearance of a voluminous dedifferentiated liposarcoma, respectively retro-peritoneal of 30 cm well encapsulated, firm with whitish areas, necrotic, slightly hemorrhagic and 20 cm hard testicle with unidentifiable spermatic cord lumen.

**Figure 3:**
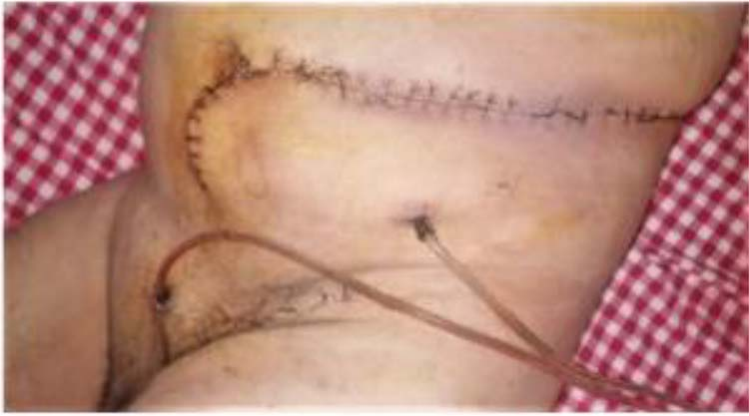
Illustrative image of wound healing with redon drains in place on postoperative day 7.

A 58-year-old man presented with an abdominal mass and swelling of the left scrotum that had been evolving for approximately three months. The patient had a good clinical appearance at the time of consultation, with no urinary or digestive complaints reported. Physical examination revealed a large, firm and painless mass occupying the entire left flank. A thoraco-abdomino-pelvic CT scan showed a voluminous retroperitoneal mass of fatty density measuring 20 × 18 × 21 cm simulating a liposarcoma, which communicates with the left inguinal canal and responsible for a left pyelocaliceal dilation (Fig. 1). Complementary inguino-scrotal MRI showed thickening of the scrotal envelopes. Resection of the retroperitoneal tumor via the intercostal approach was difficult, because it was bulky and pushed back the adjacent structures (peritoneum, spleen and left kidney). Its extraction required the opening of the peritoneum and the realization of an extended detachment up to the left colic angle and the release of the left ureter up to its crossing with the iliac vessels. Release of the lower pole of the mass revealed its extension through the left inguinal orifice, and the spermatic cord was hard on palpation. A left inguinal incision was made secondarily to extract the retroperitoneal mass en bloc with the suspect testicle and its cord (Fig. 2). Macroscopically, the mass was encapsulated weighing 4440 g, measuring 30 × 25 × 8 cm, firm, yellow-white with necrotic-hemorrhagic areas. Histology concluded to a dedifferentiated retroperitoneal liposarcoma involving the testis and its spermatic cord; the surgical histological margins were negative. The immediate postoperative evolution was normal (Fig. 3), and up to six months of follow-up.

## DISCUSSION

Retroperitoneal liposarcoma (RPLPS) is a rare and primary tumor. Its physiopathology is poorly understood. The liposarcoma (LPS) is a malignant soft tissue tumor of mesenchymal origin, developed from immature lipocytes at different stages of differentiation. It accounts for 14–18% of all malignant soft tissue tumors [4].

The disease affects, without a clear predominance of sex, the adult population of about fifty years [4, 5]. The age group of our patient (58 years) coincides with the literature. The slow increase in tumor size and the compliance of the retroperitoneal space explain the pauci-symptomatic character and the large volume of the tumor; it usually progresses to a considerable size palpable mass at the time of diagnosis [6]. The tumor only rarely shows urological signs such as low back pain, hematuria or urinary disorders, which confuses the clinician and delays the diagnosis. In most cases, it is discovered fortuitously. At the ultimate evolution, abdominal and pelvic complaints can motivate the consultation (heaviness, palpable mass or even pain). Associated urological or digestive symptoms are rare [5]. In our case, the reason for consultation was the isolated painless abdominal mass.

Inguino-scrotal invasion of the RPLPS is rarely reported in the literature. Scrotal involvement is more common with inguinal LPS than with RPLPS because the latter less often extends into the inguinal canal. In their study, Rhu et al. [2] listed only 3.6% of RPLPS cases extending into the inguinal canal. In our patient, the RPLPS invaded the left testis and its spermatic cord via the inguinal orifice. The inguinoscrotal LPS can be isolated in this area without extending into the retroperitoneal space and vice versa.

Imaging, in this case scanner and MRI, helps in diagnosis but the reference examination remains the pathological anatomy. The World Health Organization WHO, in its fifth edition 2020 of classification of soft tissue and bone tumors, classifies LPS into four subtypes, which can be compiled into three groups: myxoid LPS (56.2%), atypical LPS (well-differentiated LPS 21.9%, dedifferentiated LPS 6.8%) and pleomorphic LPS (17.8%) [7, 8]. Atypical LPS are characterized by an amplification of the MDM2 gene and CDK4, myxoid LPS by a rearrangement of the DDIT3 gene and finally pleomorphic LPS are components with a complex genome [7]. The DDLS have a strong propensity for locoregional versus distant recurrence despite advanced means of diagnosis, macroscopically complete surgical excision and possibly additional treatment. The local recurrence rate is roughly around 50% [3]. The presence of metastases is rare at the time of diagnosis. Among all histological subtypes, DDLS is high grade, faster growing and highly metastatic. The rate of metastases systemic is evaluated between 5 and 29.7% [9].

Surgical resection remains the mainstay of treatment for LPS and local recurrences [1, 2]. In the case of giant retroperitoneal liposarcoma, complete resection of the tumor and removal of invaded adjacent organs is the gold standard of the treatment. The radio chemotherapy did not show any significant effect in terms of recurrence and prognosis. According to Bonvalot et al., the rate of local relapse and metastasis is 13 vs 14%, 37 vs 29% and 50 vs 34%, respectively, after 1 year, 3 years and 5 years of appropriate surgical intervention [10]. Monitoring includes physical examination, abdominopelvic computed tomography and chest X-ray. The follow-up frequency varies according to the risk of relapse: every 3 to 6 months for 3 years, then every 6 months for 2 years and finally annually beyond 5 years [1].

## CONCLUSION

Extension of retroperitoneal liposarcoma through the inguinal canal is an extremely rare situation. Its clinical manifestation is delayed due to the compliance of the retroperitoneal space. Dedifferentiated liposarcoma has a very poor prognosis; only surgical treatment with negative margins can reduce the recurrence rate.
